# See how atoms dance

**DOI:** 10.1093/nsr/nwaa045

**Published:** 2020-03-14

**Authors:** Javier Aizpurua

**Affiliations:** Center for Materials Physics (CSIC-UPV/EHU) and DIPC, Donostia-San Sebastián, Spain

As for any other wave, classical light was often thought to be constrained by the diffraction limit. However, since the pioneering works in near-field microscopy, in which the evanescent components of light localized at the boundaries of matter beat diffraction, spectacular development of optically resolved images has been achieved in recent years, regularly obtaining nanoscale resolution of optical near-fields, as for instance in optical nanoantennas [1]. In this context, molecular atomic resolution was only within reach of tunneling electrons, able to resolve the electronic local density and obtain vibrational information on single molecules based on inelastic tunneling spectroscopy, providing a useful picturing of molecules and their dynamics [2]. The scientific community dreamt of achieving a similar capability with the use of light as a probe, but it was not until 2013 that the team by Dong and Hou at the University of Science and Technology of China, in Hefei, managed to resolve submolecular features from a single molecule with the use of Raman photons emitted from it [3]. This achievement opened up a new realm of subnanometric resolution in Optics, which is still being developed and improved [4]. After some debate, the importance of atomic-scale features, so-called ‘picocavities’, in plasmonic gaps, such as those formed in scanning tips, and in plasmonic nanoantennas has been identified as a key element to understanding of extreme optical resolution [5], which goes beyond the standard plasmonic localization.

**Figure 1.**
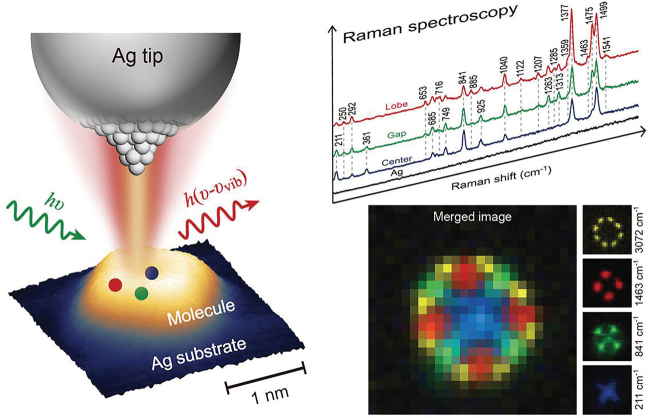
Scanning Raman picoscopy with Ångström resolution. Access to energy-filtered vibrational imaging of single molecules is possible with inelastically scattered photons from a plasmonic picocavity, enabling Ångström resolution and reconstruction of the chemical structure of the molecule.

In a new twist in molecular microscopy, the research team in Hefei has managed to combine the information obtained from a set of spectrally filtered ultra-resolved maps of Raman photons emitted from a single Mg-porphine molecule in a plasmonic cavity, and create an image of the molecule where particular vibrational modes of single bonds, as well as of ring modes, are unambiguously identified as the underlying modes sustaining a well-defined atomic structure within the molecule [6] (see Fig. 1). This is possible thanks to the combination of the aforementioned atomic resolution in the plasmonic cavity, to the relationship established between the spectrally filtered images and the vibrational database from the literature, and last, but not least, to a smart interpretation of the symmetry of the vibrational modes in the images. Taken together, this work introduces a new concept of Scanning Raman Picoscopy (SRP), as a novel tool to picture molecules by identifying their vibrational fingerprints with single chemical-bond resolution at the Ångström scale (∼1.5 Å). It seems that photons can now perform even better in a task that was meant for electrons.

An exciting prospect of this proof of principle is the combination of spectral and microscopic information obtained in this technique with the data-handling that artificial intelligence, and in particular machine learning, could offer, by properly connecting the spectral data in the literature, with the details of the energy-filtered maps in thousands of molecular species. It might be a question of time that a set of molecular images from SRP standardly determine its exact chemical composition. The monitoring of the motion of atoms in molecular assemblies might be just at hand, opening direct real-space access to the dynamics of atoms in relevant physical and chemical processes ranging from vibronic transport, to induced reactivity, through ultrasensing. Seeing them dancing is now clearer than ever.

***Conflict of interest statement***. None declared.
